# Isolation, Identification and Antimicrobial Activities of Two Secondary Metabolites of *Talaromyces verruculosus*

**DOI:** 10.3390/molecules171214091

**Published:** 2012-11-28

**Authors:** Fang Miao, Rui Yang, Dong-Dong Chen, Ying Wang, Bao-Fu Qin, Xin-Juan Yang, Le Zhou

**Affiliations:** 1 College of Life Science, Northwest A&F University, Yangling, Shaanxi 712100, China; 2 College of Science, Northwest A&F University, Yangling, Shaanxi 712100, China

**Keywords:** antimicrobial activity, isocoumarin, *Talaromyces verruculosus*, rhizosphere microorganism, *Stellera chamaejasme* L.

## Abstract

From the ethyl acetate extract of the culture broth of *Talaromyces verruculosus*, a rhizosphere fungus of *Stellera chamaejasme* L., (−)-8-hydroxy-3-(4-hydroxypentyl)-3,4-dihydroisocoumarin (**1**) and (*E*)-3-(2,5-dioxo-3-(propan-2-ylidene)pyrrolidin-1-yl)acrylic acid (**2**) were isolated and evaluated for their antimicrobial activities. Their structures were elucidated by UV, IR, MS, ^1^H-NMR, ^13^C-NMR and 2D NMR spectra. Compound **1** exhibited the significant activities *in vitro* against two strains of bacteria and four strains of fungi. Compound **2** gave slight activities on the fungi at 100 µg mL^−1^, but no activities on the bacteria. Compound **1** should be considered as a new lead or model compound to develop new isocoumarin antimicrobial agents.

## 1. Introduction

The term rhizosphere microorganism refers to a bacterial or a fungal microorganism that colonizes the region of the soil immediately adjacent (within 1 mm) to plant roots [1]. Rhizosphere microorganisms are different from those living in the nonrhizosphere surrounding soil, both in gross numbers of cells and the variety of strains. The rhizosphere microbial communities influence growth, resistance to disease or even death of the plant host depending on the degree of parasitism and pathogenicity [1,2]. The rhizosphere communities of plant species differ from each other [3,4]. The diversity of microbial strains in the rhizosphere is influenced by different plant species [5,6] and environmental factors affecting plant growth [7]. Recently, rhizosphere microorganisms have been recognized as an important source of a variety of structurally novel active secondary metabolites. As a representative example, Gunatilaka *et al. *have isolated 18 new natural products and 23 known compounds from nine strains of Sonoran desert plant-associated rhizosphere fungi [8,9,10,11,12,13,14,15]. However, compared with those of endophytes, the secondary metabolites of rhizosphere microorganisms had not received much attention.

*Stellera chamaejasme* L. (Thymelaeceae) is a perennial herb and widely distributed in North China. Its roots have been used in Chinese traditional herb medicine as an external medicament. Our previous study resulted in the isolation of a fungal strain from the rhizosphere soil of *Stellera chamaejasme* L. identified as *Penicillium verruculosum *YL-52, which was revised as *Talaromyces verruculosus* by Samson *et al.* in 2011 [16]. Meanwhile, the ethyl acetate extract of the culture broth of the fungus exhibited obvious antifungal activities against 11 strains of plant pathogens and antibacterial activities against four bacterial strains [17]. The aim of the present study was to determine the chemical constituents of the ethyl acetate extract and its active compounds. Herein, we reported the isolation and identification of two compounds from the ethyl acetate extract and their antimicrobial activities.

## 2. Results and Discussion

### 2.1. Isolation and Structural Identification

**Figure 1:**
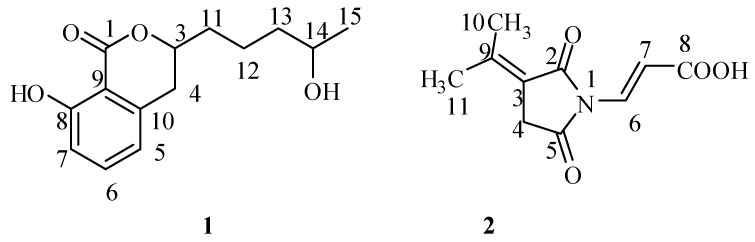
The structures of compounds **1** and **2**.

Repeated silica gel column chromatography of the ethyl acetate extract of the culture broth of the fungus YL-52, followed by purification of Sephadex LH-20 column chromatography, revealed two compounds **1** and **2** (Figure 1).

Compound **1**, isolated as white needle crystals, showed a quasi-molecular ion peak at *m*/*z* 251.1279 [M+H]^+^ in HRMS corresponding to the molecular formula C_14_H_18_O_4_. The molecular formula indicated six degrees of unsaturation within the molecule. IR spectrum revealed the presence of OH groups (3419, 1239, 1120 cm^−1^), C=O group (1657 cm^−1^) and benzene ring (3080, 1617, 1584 cm^−1^). UV absorption maxima at λ_max_ 246 nm (log*ε* 3.75) and 314 nm (log*ε* 3.56) disclosed the presence of a conjugated system. The ^1^H-NMR displayed the presence of three aromatic hydrogen atoms (*δ*_H_ 7.44, 6.92, 6.72). Based on the multiplicity and coupling constant of each peak, the three hydrogen atoms above were considered as a system of three vicinal aromatic protons. In addition, the ^1^H-NMR spectrum showed the presence of one methyl group (*δ*_H_ 1.25, *d*), two oxygenated methenyl groups (*δ*_H_4.59–4.65, 3.88) and one phenolic hydroxyl group (*δ*_H_ 11.04). In the ^13^C-NMR spectrum, fourteen carbon atom signals were observed. The DEPT spectrum showed one signal for one carbonyl carbon atom, six signals for sp^2^-hybridized carbon atoms (3 × C and 3 × CH), and seven signals for sp^3^-hybridized carbon atoms (1 × CH_3_, 2 × OCH and 4 × CH_2_). The signals at *δ*_C_ 162.0 suggested the presence of one oxygenated aromatic carbon atom.

The UV and IR data were good agreement with those published for 3-(2-hydroxypropyl)-8-hydroxy-3,4-dihydroisocoumarin isolated from the Chinese mangrove associate *Catunaregam spinosa* [18], suggesting that compound **1** contained an 8-hydroxy-3,4-dihydroisocoumarin moiety. The ^13^C-NMR spectrum was very similar to that of 3-(2-hydroxypropyl)-8-hydroxy-3,4-dihydroisocoumarin, except for the presence of two additional methylenes. Moreover, the location of the hydroxyl group at C-14 was determined by the HMBC correlations between H-15 (*δ*_H_ 1.25) and C-14 (*δ*_C_ 67.8) and between the proton of 14-OH and C-14, as well as the coupling relationship between H-15 (*δ*_H_ 1.25) and H-14 (*δ*_C_ 3.88) in ^1^H-NMR spectrum. The assignment of all proton signals in ^1^H-NMR spectrum was carried out by HSQC and HMBC correlations. Based on the facts above, **1** was established as 3-(4-hydroxypentyl)-8-hydroxy-3,4-dihydroisocoumarin. The optical rotation ([*α*]_D_^25^ −40.4 (*c* 0.19, acetone)) showed that **1** was an optically pure chiral compound, but the configurations at C-3 and C-14 remained unsolved because the crystal of **1** was too fragile for an X-ray crystallographic determination. A similar case was observed for 3-(2-hydroxypropyl)-8-hydroxy-3,4-dihydro-isocoumarin [17].

Compound **2** was obtained as a white powder, and established as (*E*)-3-(2,5-dioxo-3-(propan-2-ylidene)pyrrolidin-1-yl) acrylic acid by X-ray crystallographic analysis in our previous study [18]. Herein, we further reported its other previously unpublished spectroscopic data. Compound **2** possessed a molecular formula of C_10_H_11_NO_4_, as deduced from its quasi-molecular ion peak at *m*/*z* 210.0762 [M+H]^+^ in HR-ESI-MS. The molecular formula indicated six degrees of unsaturation. The IR spectrum revealed the presence of an OH group (3431, 1234 cm^−1^), C=O groups (1714, 1628 cm^−1^) and CH_3_ groups (2924, 1359 cm^−1^); The UV disclosed absorption maxima at λ_max_ 262 nm (log*ε* 4.01), due to the presence of a conjugated system; The ^1^H-NMR spectrum exhibited the presence of two methyl groups at *δ*_H_ 2.35 (3H, *s*) and 1.93 (3H, *s*), two olefinic protons at *δ* 7.78 (1H, *d*) and 6.91 (1H, *d*), and one methylene group at *δ*_H_ 3.35 (2H, *s*). The above findings were further supported by the DEPT spectrum. The ^13^C-NMR spectrum displayed 10 signals, of which five were assigned to quaternary carbon atoms by DEPT. Based on the large coupling constant of the signals at *δ*_H_7.78 (1H, *d*, *J* = 14.8) and 6.91 (1H, *d*, *J* = 14.8), a *trans* configuration was established for the ∆^6^-double bond. In the HMBC spectrum, the correlations between H-6 and C-2, C-5, C-8 suggested that the CH=CHCO_2_H group was attached to a nitrogen atom, and the correlations between H-10 and C-3, C-9, C-11 and between H-11 and C-3, C-9, C-10 suggested the occurrence of one propan-2-ylidene moiety at the C-3 position. From the foregoing, it was concluded that compound **2** was (*E*)-3-(2,5-dioxo-3-(propan-2-ylidene)pyrrolidin-1-yl) acrylic acid, in agreement with its X-ray crystallographic analysis result [19].

### 2.2. Bioactivity

The antimicrobial activities of **1** and **2** were determined against pathogenic bacteria and fungi as listed in Table 1. The results showed that **1** had obvious antibacterial activities against *Staphylococcus aureus* and *Escherichia coli*, with minimum inhibitory concentration (MIC) values of 2.5 µg mL^−1^ and 5.0 µg mL^−1^, respectively, but **1** was less active than the standard drug ceftriaxone sodium (a broad-spectrum antibiotic) with an MIC value of 0.625 µg mL^−1^. For plant pathogenic fungi, **1** disclosed significant growth inhibitions of 92.6 ± 2.1%, 97.3 ± 3.3%, 87.2 ± 2.8% and 94.9 ± 1.9% at 50 µg mL^−1^ against *Alternaria solani*, *Valsa mali*, *Curvularia lunata *and *Botryosphaeria berengeriana*, respectively. Under the same conditions, the growth inhibitions of thiabendazole, a broad-spectrum antifungal drug, were 54.7 ± 2.6%, 100 ± 0.0%, 51.5 ± 4.1, compound **2** was inactive for both the tested bacteria and fungi, although it showed slight antifung% and 92.8 ± 3.7%. Obviously, **1** had higher activities than thiabendazole against phytopathogenic fungi. Under the same conditions al activities at a concentration of 100 µg mL^−1^.

**Table 1:** *In vitro* antimicrobial activities of **1** and **2** (50 µg mL^−1^).

Compound	Growth inhibition (%, mean ± SD)				MIC (µg mL^−1^)	
	*A. solani*	*V. mali*	*C. lunata*	*B. berengeriana*	*S. aureus*	*E. coli*
**1**	92.6 ± 2.1	97.3 ± 3.3	87.2 ± 2.8	94.9 ± 3.9	2.5	5.0
**2**	N ^a^ (16.3 ± 5.4) ^b^	N (21.3 ± 4.6)	N (25.6 ± 5.4)	N (19.7 ± 3.8)	>100	>100
**Thiabendazole**	54.7 ± 2.6	100 ± 0.0	51.5 ± 4.1	92.8 ± 3.7	―	―
**Ceftriaxone sodium**	―	―	―	―	0.625	0.625

^a^: N: No inhibition of growth was observed at 50 µg mL^−1^; ^b^: The numbers in parentheses were growth inhibition rates at 100 µg mL^−1^.

## 3. Experimental

### 3.1. General

Optical rotations: Horiba SEPA-300 digital polarimeter. IR spectra: Nexus FT-IR 400 spectrometer; with KBr pellets; in cm^−1^. UV spectra: SP-2100 UV/VIS spectrometer; λ_max_ (logε) in nm. NMR spectra were recorded on Bruker AV-400 spectrometers with TMS as an internal standard. MS spectra were recorded with a VG Autospec-3000 spectrometer. HR-ESI-MS were recorded with an API QSTAR Pulsar 1 spectrometer; in *m*/*z*. Column chromatography was carried out on silica gel (200–300 mesh, Qingdao Marine Chemical Inc., Qingdao, China) and Sephadex LH-20 (Pharmacia, New Jersey, NJ, USA). Fractions were monitored by TLC and spots were visualized by heating silica gel plates sprayed with 10% H_2_SO_4_ in ethanol. 

### 3.2. Plant and Fungal Strain Materials

The fungal strain was isolated in the rhizosphere soil of *Stellera chamaejasme* L. collected in the Qinling Mountains of Taibai town in Shaanxi Province, China, in August 2007 and deposited in the Laboratory of Natural Product Research, Northwest A&F University, Shaanxi Province, China (culture collection number YL-52). The fungus was identified as *Penicillium verruculosum *YL-52 (the revised name: *Talaromyces verruculosus*) by the streak plate method, morphological characteristics, sequence analysis of ITS and comparison analysis with GenBankB data [16]. 

### 3.3. Fermentation

Cultures of YL-52 maintained on potato-dextrose-agar (PDA) medium slants were subcultured in Petri dishes prior to testing. Two fungal cakes (*d* = 13 mm) were added to a 250 mL conical flask containing 100 mL potato-dextrose broth (PDB), and cultured for 9 d at 25 °C with a 150 r/min frequency in a rocking bed. A 60 L culture broth was obtained from 600 fermentation flasks.

### 3.4. Extraction and Isolation

The culture broth (60 L) was extracted with ethyl acetate (50 L × 3). The combined ethyl acetate solution was concentrated under vacuum to yield 12.8 g of crude extract. Ten grams of the extract were subjected to column chromatography over silica gel using CHCl_3_-CH_3_OH (stepwise gradient: 100:1, 50:1, 25:1, 10:1, 5:1, 1:1, 0:1, v/v) to furnish the corresponding fractions A-1 (0.8 g), A-2 (3.80 g), A-3 (1.44 g), A-4 (1.21 g), A-5 (0.32 g), A-6 (0.30 g) and A-7 (1.95 g). Fractions A-2 and A-3 were combined and subjected to column chromatography over silica gel using petroleum ether-EtOAc (stepwise gradient: 20:1, 15:1, 10:1, 5:1, 1:1, 0:1; petroleum ether: b.p. 60–90 °C) to obtain corresponding subfractions B-1 (0.57 g), B-2 (0.76 g), B-3 (0.97 g), B-4 (0.86 g), B-5 (0.94 g) and B-6 (0.90 g). Subfractions B-3 and B-4 were combined, and subjected to column chromatography on SiO_2_ with petroleum ether-acetone (stepwise gradient: 10:1, 8:1, 5:1, 2:1, 1:1, 0:1). The petroleum ether–acetone (5:1) eluate was sequentially subjected to preparative silica gel TLC using petroleum ether–acetone (2:1) and Sephadex LH-20 column chromatography with methanol. Compound **1** (16 mg) was obtained as colorless needle-like crystals (petroleum ether–EtOAc, 1:1). Fractions A-5 and A-6 were combined and repeatedly subjected to column chromatography over silica gel using petroleum ether–acetone mixture (stepwise gradient: 5:1, 2:1, 1:1, 0.5:1). The petroleum ether-acetone (0.5:1) eluate was purified by preparative silica gel TLC (CHCl_3_–CH_3_OH, 1:1）and Sephadex LH-20 column chromatography using methanol to provide **2** (14 mg) as a white powder.

*3-(4-Hydroxypentyl)-8-hydroxy-3,4-dihydroisocoumarin* (**1**)*. *White needle-like crystals (petroleum ether–ethyl acetate), *R*_f_ = 0.35 (petroleum ether–acetone, 2:1), 0.56 (CHCl_3_–CH_3_OH, 8:1), m.p. 80–81 °C, [*α*]_D_^25^ −40.4 (*c* 0.19, acetone); UV (95% EtOH): λ_max_ (log*ε*) = 212 (4.30), 246 (3.75), 314 (3.56) nm; IR (KBr): *ν*_max _= 3419, 3080, 2946, 1657, 1617, 1584, 1462, 1239, 1120 cm^−1^; ^13^C-NMR (100 MHz, CDCl_3_) *δ* ppm: 169.8 (C-1), 162.0 (C-8), 139.3 (C-10), 136.1 (C-6), 117.9 (C-5), 116.1 (C-7), 108.3 (C-9), 79.6 (C-3), 67.8 (C-14), 38.7 (C-13), 34.7 (C-11), 32.8 (C-4), 23.6 (C-15), 21.1 (C-12);^ 1^H-NMR (400 MHz, CDCl_3_) *δ* ppm: 11.04 (1H, *s*, 8-OH), 7.44 (1H, *t*-like, *J* = 8.0 Hz, H-6), 6.92 (1H, *d*, *J* = 8.4 Hz, H-7), 6.72 (1H, *d*, *J* = 7.6 Hz, H-5), 4.59-4.65 (1H, *m*, H-3), 2.96-2.99 (2H, *m*, H-4), 1.89–2.00 (1H, *m*, H-11a), 1.77-1.85 (1H, *m*, H-11b), 1.60–1.77 (2H, *m*, H-12), 1.50–1.56 (2H, *m*, H-13), 3.88 (1H, *hepta*, *J* = 6.4, 11.6, 17.6 Hz, H-14), 1.25 (3H, *d*, *J* = 6.4 Hz, H-15), 1.69 (1H, *s*, 14-OH); Main HMBC correlations: *δ*_H_ 2.96–2.99 (H-4) / *δ*_C_ 79.6 (C-3), 117.9 (C-5), 108.3 (C-9), 139.3 (C-10); *δ*_H_ 6.72 (H-5) / *δ*_C_ 32.8 (C-4), 108.3 (C-9); *δ*_H_7.44 (H-6) / *δ*_C_ 116.1 (C-7), 139.3 (C-10); *δ*_H_ 6.92 (H-7) / *δ*_C_ 117.9 (C-5), 108.3 (C-9); *δ*_H_1.89-2.00 (H-11a), 1.77-1.85 (H-11b) / *δ*_C_ 79.6 (C-3); *δ*_H_ 1.50–1.56 (H-13) / *δ*_C_ 23.6 (C-15); *δ*_H_ 1.25 (H-15) / 67.8 (C-14), 38.7 (C-13); *δ*_H_1.69 (14-OH) / 67.8 (C-14), 23.6 (C-15); (+)-ESI MS *m*/*z* = 251.0 [M+H]^+^; (+)-ESI HR MS *m*/*z* = 251.1279 [M+H]^+^ (calcd. for C_14_H_19_O_4_, 251.1283). 

*(E)-3-(2,5-Dioxo-3-(propan-2-ylidene)pyrrolidin-1-yl)acrylic acid *(**2**)*. *White powders, *R*_f_ = 0.54 (chloroform–methanol–EtOAc, 2:3:3), 0.76 (chloroform–methanol, 1:1), m.p. 194–195 °C; UV (95%EtOH): λ_max_ (log*ε*) = 262 (4.01) nm; IR (KBr): *ν*_max _= 3431, 2924, 1714, 1628, 1359, 1234 cm^−1^; ^13^C-NMR (100 MHz, CDCl_3_) *δ* ppm: 173.9 (C-8), 170.9 (C-5), 168.3 (C-2), 154.1 (C-9), 133.2 (C-6), 119.3 (C-3), 110.9 (C-7), 48.6 (C-4), 24.4 (C-11), 21.0 (C-10); ^1^H-NMR (400 MHz, CDCl_3_) *δ* ppm: 7.78 (1H, *d*, *J* =14.8 Hz, H-6), 6.91 (1H, *d*, *J* = 14.8 Hz, H-7), 3.35 (2H, *s*, H-4), 2.35 (3H, *s*, H-10), 1.93 (3H, *s*, H-11); Main HMBC correlations: *δ*_H_ 7.78 (H-6) / *δ*_C _173.9 (C-8), 170.9 (C-5), 168.3 (C-2); *δ*_H_ 6.91 (H-7) / *δ*_C _133.2 (C-6); *δ*_H_ 2.35 (H-10) / *δ*_C _154.1 (C-9), 119.3 (C-3), 24.4 (C-11); *δ*_H_ 1.93 (H-11) / *δ*_C _154.1 (C-9), 119.3 (C-3), 21.0 (C-10); (+)-ESI MS *m*/*z* = 209.9 [M+H]^+^; (+)-ESI HR MS *m*/*z* = 210.0762 [M+H]^+^ (calcd. for C_10_H_12_NO_4_, 210.0766).

### 3.5. Antibacterial Assay

The antibacterial activity was determined by method reported in the literature [20]. A LB (Luria-Bertani) broth (50 mL) was used for culturing *E. coli* and *S. aureus*. Half milliliter of the 24 h cultured fresh broth at 37 °C was diluted by 50 mL of the same broth to indicate a 0.5 McFarland (McF) turbidity (ca. 1.5 × 10^8^colony forming unit). A tested compound was dissolved in dimethylsulfoxide (DMSO) to provide a sample solution of 0.8 mg mL^−1^, and then double-fold serial dilutions (0.625–160 μg mL^−1^) were made by adding Mueller-Hinton broth. Each sample solution (100 μL) was completely mixed with the bacterial suspension (100 μL) in a well of 96-well polystyrene plate. After incubation of the plates for 24 h at 37 °C, the absorbance of each well was measured at 630 nm with a microtiter plate reader (BioRad). According to the same, were prepared and used as positive control. Five percent DMSO in the same broth served method as the tested compounds, the double-fold serial solutions of ceftriaxone sodium (0.156–2.50 μg mL^−1^), a broad spectrum antibiotics as negative control. All experiments were performed in triplicate. The minimal inhibitory concentration (MIC, μg mL^−1^) was defined as the concentration of a compound required to reduce the absorbance to less than 50% of that of the negative control.

### 3.6. Antifungal Assay

The antifungal activity *in vitro* was assayed by the growth rate method [21] with slight modifications. The tested pathogenic fungi were *Alternaria solani*, *Valsa mali*, *Curvularia lunata *and *Botryosphaeria berengeriana*, provided by the Institute of Pesticides, Northwest A&F University. Cultures of the test fungi maintained on potato-dextrose-agar (PDA) medium slants were subcultured in Petri dishes prior to testing. Sample solution (1 mg mL^−1^) in acetone was completely mixed with the autoclaved PDA medium to provide the medium containing 50 µg mL^−1^ of sample, and poured into Petri dishes. Five percent acetone in PDA medium served as negative control. PDA medium containing 50 (or 100) µg mL^−1^ of thiabendazole, a broad-spectrum antifungal drug, was used as positive control. When the medium in the plates was partially solidified, a 5-mm thick and 4 mm diameter disc of fungus cut from earlier subcultured Petri dishes was placed at the centre of the semi-solid medium. The treated and control dishes were kept in an incubator at 26 (±2) °C for 72 h. The diameters (in mm) of inhibition zones were measured in three different directions. Growth inhibition rates were calculated according to the following formula and expressed as means ± SD:

%Growth inhibition rate = (*d*_c_ − *d*_s_) / (*d*_c_ − *d*_0_) × 100

where *d*_0_: Diameter of the fungus cut, *d*_c_: Diameter of the untreated control fungus, *d*_s_: Diameter of the sample-treated fungus. 

## 4. Conclusions

In conclusion, the present study described the isolation, characterization and antimicrobial activities of two secondary metabolites **1** and **2** of *Talaromyces verruculosus* isolated in the rhizosphere soil of *Stellera chamaejasme* L. and reported their UV, IR, MS, 1D and 2D NMR spectroscopic data for the first time. Compound **1** showed significant antimicrobial activities against four strains of fungi (*A. solani*, *V. mali*, *C. lunata*, * B. berengeriana*) at 50 µg mL^−1^ and two strains of bacteria (*S.* *aureus* and *E. coli*) with MIC values of 2.5 µg mL^−1^ and 5.0 µg mL^−1^, respectively. Compound **2** was slightly active against the fungi at 100 µg mL^−1^and its MIC values on the bacteria were more than 100 µg mL^−1^. Compound **1** should be considered as a new lead or model compound to develop new isocoumarin antimicrobial agents.
